# Neovascularization in Atherosclerotic Plaques: Clinical Implications, Detection, and Prevention Strategies

**DOI:** 10.31083/RCM36468

**Published:** 2025-07-23

**Authors:** Long Zhang, Chancui Deng, Sha Wang, Bei Shi, Guanxue Xu

**Affiliations:** ^1^Department of Cardiology, Zunyi Medical University, 563003 Zunyi, Guizhou, China; ^2^Department of Cardiology, Affiliated Hospital of Zunyi Medical University, 563003 Zunyi, Guizhou, China; ^3^Department of Cardiology, The Fifth Affiliated Hospital (Zhuhai) of Zunyi Medical University, 519000 Zhuhai, Guangdong, China

**Keywords:** neovascularization, atherosclerotic plaques, clinical implications, detection techniques, prevention strategies

## Abstract

Following the global increase in atherosclerotic cardiovascular diseases, the demand for the effective identification of high-risk factors that lead to atherosclerotic plaque rupture and the search for new therapeutic targets has also increased. Neovascularization within plaques is widely recognized as an important indicator of plaque vulnerability. Thus, the timely detection of neovascularization within plaques and early intervention treatment can help reduce the potential adverse cardiovascular events caused by plaque rupture. This article introduces the formation mechanism, clinical significance, detection techniques, and prevention strategies for neovascularizing atherosclerotic plaques.

## 1. Introduction

The number of people suffering from cardiovascular diseases is increasing globally. In 1990, it was estimated that more than 271 million people were affected with cardiovascular disease. By 2019, this figure had increased to 523 million people. Atherosclerotic diseases, particularly ischemic heart disease and stroke, are the primary factors contributing to the burden of cardiovascular disease and driving its developmental trends [1]. Atherosclerosis is a vascular inflammatory disease caused by plaque accumulation and is characterized by local thickening of the vascular wall and plaque formation [2]. Although there are usually no symptoms in the early stages, as the disease progresses, the plaque may become unstable, leading to serious acute cardiovascular events. When a plaque ruptures and blocks the coronary or carotid arteries, it may lead to myocardial infarction, transient ischemic attack, and stroke [3]. The characteristics of these unstable plaques mainly include thin fibrous caps, large lipid nuclei, plaque bleeding, inflammation, and obvious neovascularization [4]. The neovascularization within the plaque is regarded as the small blood vessels that grow into the plaque from the vasa vasorum of the adventitia after stimulation, and they play a promoting role in the growth of atherosclerotic plaques [5].

Many studies have linked neovascularization with vulnerable plaques and revealed its significance as a reliable prognostic indicator of high-risk vulnerable plaques [6, 7, 8, 9, 10, 11, 12]. Some research groups have also considered neovascularization in plaques as a potential target to slow down or reverse the progression of atherosclerotic diseases [13, 14, 15, 16, 17]. These findings emphasize the importance of in-depth studies of neovascularization in cardiovascular health management.

## 2. Mechanism of Neovascularization in Plaque

Neovascularization usually originates from the nutrient vessels in the vascular wall, which are located between the tunica media and tunica adventitia [18]. When pathological factors that stimulate angiogenesis are present, nutrient vessels become activated and extend deep into the vascular intima or plaque to continue growing [19]. Currently, some of the known pathological factors include hypoxia, lipids, and inflammation. Hypoxia in the plaque environment is an effective inducer of neovascularization [8]. In fact, hypoxia promotes the activation of hypoxia inducible factor 1 (HIF1), which initiates the transcription of angiogenic factors such as vascular endothelial growth factor A (VEGF-A) and E26 transformation related factor-1 (Ets-1). Ets-1 is a transcription factor that activates VEGF expression. Simultaneously, VEGF promotes the activity of Ets-1. The induction and activation of VEGF and Ets-1 provides support for neovascularization [20]. Secondly, neovascularization in the plaque is regarded as a pathway for lipids to enter the plaque. The activation/proliferation of medial smooth muscle cells may be caused not only by hypoxia but also by neovascularization or by lipids accumulated in the necrotic core. These lipids and their derivatives can act as activators of peroxisome proliferator-activated receptor γ (PPARγ) in smooth muscle. PPARγ activation promotes smooth muscle cells to generate VEGF-A, thereby facilitating neovascularization [21]. A cross-sectional study from 2018 to 2019 in China [22] showed that the increase in total cholesterol (TC), low-density lipoprotein cholesterol (LDL-C), and non-high-density lipoprotein cholesterol (non-HDL-C) was related to the increase in the detection rate of neovascularization in carotid plaques in the stroke risk population. Lastly, it has also been pointed out that neovascularization is usually formed in the intima where inflammatory immune cells accumulate chronically [23]. The inflammatory cells in the plaque increase the oxygen demand, which leads to further neovascularization. In addition, with the progress of neovascularization, its inherent leakage and increase in adhesion molecules cause more inflammatory cells to gather in the plaque. Activated inflammatory cells secrete various cytokines, growth factors, and angiogenic factors [24], thus stimulating the formation of new blood vessels. The interaction between inflammation and neovascularization further aggravates the complexity of atherosclerotic plaques.

In conclusion, the formation of neovascularization is closely related to nutrient vessels within the vascular wall. When local stimuli such as hypoxia, lipids, and inflammation are present, the nutrient vessels can be activated and grow towards the vascular endothelium or into the plaque, ultimately forming neovascularization within the plaque. Within atherosclerotic plaques, neovascularization is mainly present in the shoulder regions, and it is more common in the upstream shoulder than in the downstream shoulder. Neovascularization increases with plaque progression, and its content is significantly higher in advanced lesions than in early lesions [25]. A deeper investigation of the interplay between neovascularization, hypoxia, and inflammatory responses may provide new directions for the treatment of atherosclerosis. Targeting VEGF, Ets-1, and their associated pathways to reduce inflammation and neovascularization within the plaque promises to improve atherosclerosis prognosis [26]. Additionally, monitoring the development of neovascularization in conjunction with biochemical indicators (such as cholesterol levels) is also important for early diagnosis and intervention of the disease. This research area has the potential to provide more comprehensive treatment strategies that contribute to improving overall cardiovascular health.

## 3. Effect of Neovascularization in Plaque on Plaque Stability

Intraplaque hemorrhage is one of the most dangerous characteristics of plaque rupture, which is closely related to the occurrence of neovascularization in plaque rupture [27]. However, these neovascularization lesions are usually immature, very fragile, and tortuous, and their endothelial cell connections are weak and prone to leakage and rupture [28]. When there are changes in the hemodynamics of blood flow inside the lumen of blood vessels and in the external environment, neovascularization within the plaque is usually affected, leading to impaired function. As a result, red blood cells and inflammatory cells within it leak into the plaque, causing intraplaque hemorrhage and aggregation of inflammatory cells, which may reduce the stability of plaques, ultimately leading to the occurrence of adverse cardiovascular events. Bleeding in plaques is thought to be caused by the leakage of red blood cells from dysfunctional neovascularization, and these red blood cell membranes are rich in free cholesterol [29]. The accumulation of cholesterol crystals in blood vessels leads to the expansion of the necrotic core and, through the stimulation of the nucleotide-binding oligomerization domain-like receptor family, pyrin domain-containing 3 (NLRP3) inflammasome pathway, and interactions with cells such as macrophages, lymphocytes, and neutrophils, induces the production of interleukin 1β (IL-1β), thereby further exacerbating the inflammation of the necrotic core [30]. In addition, cholesterol crystallization can also lead to rupture of the fiber cap through mechanical puncture [31]. Therefore, intraplaque hemorrhage is an important finding in unstable atherosclerotic plaque. In addition, inflammatory cells such as macrophages, mast cells, and T cells exist in the neovascularization of plaques. They can leak from neovascularization with damaged structural integrity and accumulate around them. These inflammatory cells are rich sources of cytokines, growth factors, cysteine proteinases, and extracellular matrix metalloproteinases (MMP) [32]. Some cytokines, including interferon-γ, have a great impact on the fibrous cap of plaques. It is a cytokine produced by Th1 type T cells and NK cells to promote inflammation and activate macrophages, which can inhibit the formation of collagen fibers, resulting in increased plaque vulnerability and decreased collagen fiber content [33]. Activated macrophages produce extracellular MMP and cysteine proteases through myeloperoxidase. The collagen solubility of these two enzymes can thin the fibrous cap of the plaque, which may eventually lead to plaque rupture [34]. Since neovascularization can indirectly lead to the production of MMP and cysteine proteases in plaques, there is a direct connection between neovascularization and plaque rupture.

In summary, neovascularization undermines plaque stability and increases the risk of cardiovascular events, primarily through intraplaque hemorrhage and the concomitant accumulation of inflammatory cells. Future research must further elucidate the precise mechanisms connecting neovascularization with intraplaque hemorrhage, and examine how inflammatory cells and their secreted factors influence plaque architecture and functionality. Moreover, fostering targeted therapeutic approaches to enhance plaque resilience and manage intraplaque hemorrhage could significantly diminish the prevalence of cardiovascular events. Exploring interventions that target cholesterol crystals and inflammasomes is crucial for facilitating the early detection and intervention of unstable atherosclerotic plaques. Taruya* et al*. [35] conducted a study on neovascularization in different plaque types. They quantified neovascularization within the plaques and compared it among different types of plaques. Finally, the research results showed that neovascularization was more common in ruptured plaques, followed by fibroatheromas, and less common in fibrous plaques and fibrocalcific plaques. This further demonstrates that neovascularization is more common in advanced and unstable plaques.

## 4. Correlation Between Neovascularization in Plaque and Adverse Cardiovascular Events

Coronary heart disease is one of the most serious threats to human health worldwide. Percutaneous coronary intervention (PCI) is currently the main treatment method for patients with severe coronary heart disease. Although revascularization therapy has significantly improved the survival rate of patients with coronary heart disease, the occurrence of major adverse cardiovascular events (MACE) after treatment remains inevitable [36]. Hou *et al*. [37] conducted a median follow-up of 620 days for patients who received revascularization treatment, the research results showed that the incidence of MACE after revascularization treatment was as high as 35.46%. According to previous experience and cognition, maximum plaque thickness and degree of vascular stenosis are generally considered to be the main risk factors for cardiovascular events [38]. However, recent studies have shown that hemodynamic changes in many plaque lesions leading to adverse cardiovascular events before plaque rupture or thrombosis are not significant, and that there are no related ischemic symptoms [39]. The study by Virmani *et al*. [40] showed that the number of neovascularization lesions in vulnerable plaques increased by two times, while the number of neovascularization lesions in ruptured plaques increased by four times, compared with stable plaques with severe lumen stenosis. Staub *et al*. [41] found that there was no obvious correlation between the neovascular density in plaques and traditional cardiovascular risk factors, but there was a certain relationship with patients who had experienced myocardial infarction, indicating that neovascular density may be an independent predictor of plaque vulnerability. Multivariate analysis showed that the presence of neovascularization in plaques was the most important marker associated with previous cardiovascular events, and its correlation even exceeded traditional risk factors such as age, hypertension, diabetes, or smoking. van der Toorn *et al*. [42] demonstrated that intraplaque hemorrhage in the carotid artery is a powerful predictor of atherosclerotic cardiovascular disease in women. This predictive effect is independent of traditional cardiovascular risk factors, other plaque components, plaque size, and the degree of vascular stenosis. However, this correlation was not as strong in male patients. The probability of cardiovascular events increases with an increase in women’s age. It is speculated that this may be related to the decrease in estrogen levels in women with age. Combined with the previous discussion, it has been pointed out that intraplaque hemorrhage is caused by rupture of neovascularization within the plaque. Therefore, it is concluded that the formation of neovascularization within the plaque has sex specificity in relation to the occurrence of cardiovascular events. These findings indicate that neovascularization in plaques is expected to become a marker of plaque vulnerability in healthy individuals with subclinical atherosclerosis. We collected relevant studies on neovascularization and cardiovascular events and summarized them in Table 1 (Ref. [43, 44, 45, 46]).

**Table 1.** **Correlation study on the relationship between neovascularization and adverse cardiovascular and cerebrovascular risk factors**.

Year	Content	Author	Reference
2018	Compared to patients with neovascularization within plaques, those without neovascularization had a larger mean minimum lumen area (2.74 mm^2^ vs 2.45 mm^2^; *p* = 0.036), fewer vulnerable plaques (56.8% vs 47.6%; *p* = 0.019), and a smaller plaque burden (50.7% vs 68.2%; *p* = 0.035).	Xu *et al*.	[43]
2019	From baseline to follow-up, a significant increase was observed in plaque area percentage, plaque plus media cross-sectional area, plaque volume, and plaque burden in the neovascularization group. Despite receiving statin therapy, neovascularization within the plaque remained a high-risk predictor of plaque progression [OR 6.521 (95% CI 2.457–17.308), *p * < 0.001].	Liu *et al*.	[44]
2019	Higher scores of intraplaque neovascularization assessed by carotid artery contrast-enhanced ultrasound (CEUS) are associated with significant coronary artery disease (≥50% stenosis) (1.8 ± 0.4 vs 0.5 ± 0.6, *p * < 0.0001) and greater complexity of coronary lesions (1.7 ± 0.5 vs 1.3 ± 0.8, *p * < 0.0001).	Mantella *et al*.	[45]
2023	In patients with in-stent restenosis and neovascularization, the incidence of neoatherosclerosis is higher than that in patients without neovascularization (*p * < 0.001). Patients with both in-stent restenosis and neovascularization also have higher rates of macrophage infiltration, thin-cap fibroatheroma, plaque rupture, and thrombosis (*p * < 0.01).	Deng* et al*.	[46]

OR, odds ratio.

## 5. Contemporary Detection Technology of Neovascularization in Plaque

The close relationship between vulnerable atherosclerotic plaques and cardiovascular events makes the detection and diagnosis of vulnerable plaques an important research topic. Neovascularization of plaques is one of the main characteristics of vulnerable plaques. By detecting the formation of neovascularization in plaques, vulnerable atherosclerotic plaques can be detected and prevented early in the diagnosis and prevention of acute cardiocerebral ischemic events [9]. At present, there are mainly two kinds of detection and diagnosis techniques for neovascularization in plaque: invasive and non-invasive.

### 5.1 Invasive Detection Technology of Neovascularization in Plaque

Intravascular medical invasion imaging is a method of collecting images from the inside of blood vessels by using a special catheter with a small probe. These methods have a higher image resolution than non-invasive alternatives [47]. It plays a crucial role in the diagnosis and study of cardiovascular diseases in modern medicine. This imaging modality can obtain a higher resolution than non-invasive methods because its probes can penetrate directly into the blood vessels and provide a closer view of the vessel wall and lumen. Intravascular ultrasound (IVUS), optical coherence tomography (OCT), and near-infrared fluorescence (NIRF) are the latest invasive imaging techniques used to evaluate neovascularization in plaques [48]. In addition, OCT and IVUS are the most commonly used intracavitary imaging techniques.

#### 5.1.1 IVUS

IVUS uses ultrasound technology to generate cross-sectional images of the large vascular lumen and wall with a resolution of 100 µm. IVUS has remarkable unique advantages, with its tissue penetration depth reaching up to 5–6 millimeters. IVUS is capable of observing deeper vascular structures and can even visualize the adventitia, especially in large blood vessels or when the plaque burden in the blood vessels increases [49]. In clinical practice, IVUS measures the external elastic membrane and uses the smallest external elastic membrane diameter in the reference area as key data, providing precise references for the selection of stent sizes [50]. This method ensures that the selected stent size fits the blood vessel, greatly reducing the probability of complications such as coronary artery dissection, perforation, extensive malapposition, and inadequate expansion. Although IVUS can provide stable detection of trophoblastic vessels, it has certain resolution limitations in the accurate identification of plaque phenotypes [51]. Taking the study of Vavuranakis and others [52] as an example, they tried to use contrast-enhanced IVUS to evaluate the neovascular density in coronary atherosclerotic plaques in patients with acute myocardial infarction and diabetes mellitus, but they could only observe the gray intensity enhancement of intima-media and adventitia, and could not directly observe the neovascularization in detail. In contrast, IVUS enhanced with a contrast agent can observe neovascularization in plaques with high resolution, which provides the possibility for more accurate evaluation; in the future, contrast-enhanced IVUS technology can be further optimized to improve its accuracy and utility in assessing plaque neovascularization in clinical practice. This may help diagnose atherosclerosis-related diseases more accurately and provide a more reliable basis for the development of personalized treatment plans. Meanwhile, combining it with other diagnostic techniques could provide a more comprehensive understanding of vascular lesions to better guide treatment and predict disease progression.

#### 5.1.2 OCT

**Fig. 1.**
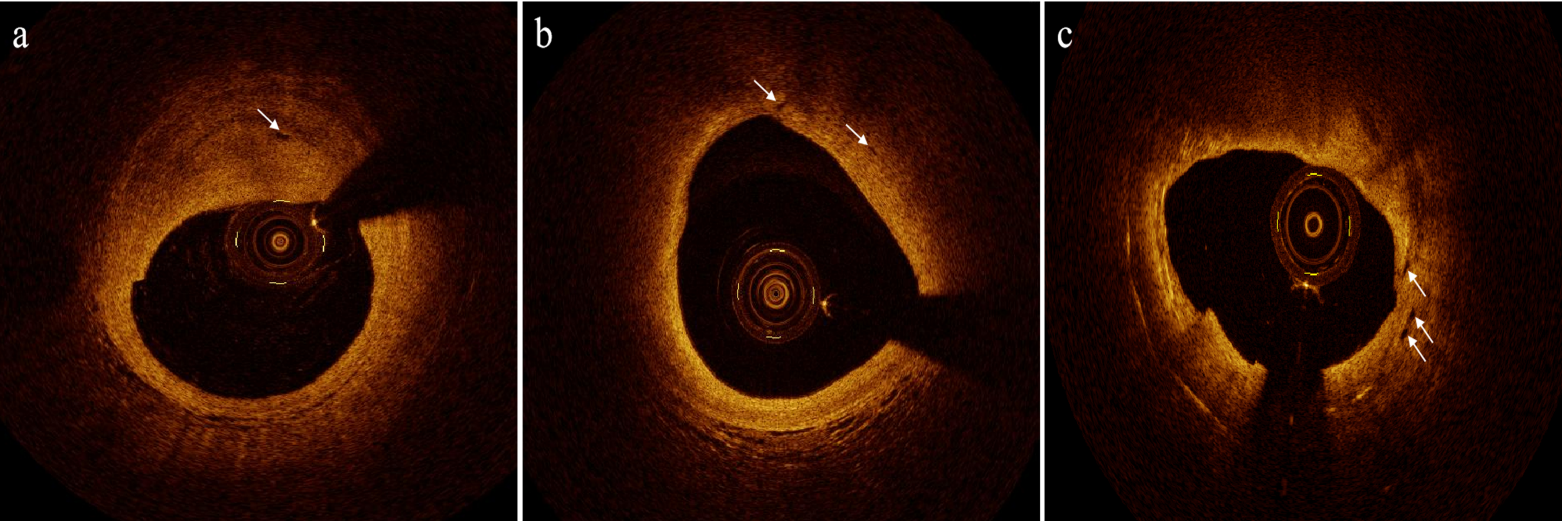
**Representative optical coherence tomography (OCT) images of neovascularization**. (a) Fibrous plaque with neovascularization (white arrow). (b) Lipid plaque with neovascularization (white arrow). (c) In-stent thin-cap fibroatheroma with neovascularization (white arrow). (The OCT images were obtained using the ILUMIEN OPTIS intravascular imaging system (St. Jude Medical, Saint Paul, MN, USA). The images were post-processed with https://ps.gaoding.com/#/.)

OCT uses near-infrared light to generate cross-sectional intravascular images, with a resolution of 10–20 µm. Compared to IVUS, OCT has a higher resolution, which enables it to provide finer images [53]. This imaging technique is based on the principle of optical interference. It uses low-coherence light interference to obtain information about the microstructure of biological tissues. When the light from a light source is divided into reference light and sample light, the reference light is reflected by a known mirror, and the sample light hits the biological tissue and reflects back. These two beams of light interfere with the detector, and an interference signal is generated only when the difference in their optical ranges is within the coherence length of the light source. By scanning the sample light at different locations in the tissue, information regarding the reflected light at different depths within the tissue can be obtained, and a tomographic image of the tissue can be constructed [54]. Therefore, based on the characteristics of high-resolution OCT, it can be used in clinical applications to evaluate neointimal hyperplasia, neoatherosclerosis, uncovered stents, stent malapposition, etc., which are associated with adverse cardiovascular events, thus improving the prognosis of patients [55]. Neovascularization is well defined in the field of medical diagnostics. Specifically, when tissue is examined by OCT, small vesicles or tubular structures are recognized as neovascular if they can be observed on at least three consecutive cross-sectional OCT images [46]. OCT has excellent performance in the visualization of neovascular structures in coronary atherosclerotic plaques and can accurately evaluate the fine neovascular branches in coronary artery lesions [56]. This makes OCT a technology to accurately detect neovascularization in plaques *in vivo* and provides a powerful tool for in-depth understanding of atherosclerotic lesions. However, OCT has some limitations. Owing to the low tissue penetration depth of OCT (1–2 mm), the blood vessel wall cannot be fully visualized, especially in the case of lipid-rich plaques and red thrombi. Due to this shortcoming, neovascularization in the deep layers of blood vessels and at the base of plaques, as well as other plaque characteristics, may be underestimated [57]. The representative OCT images of neovascularization are shown in Fig. 1.

In 2018, Sheth *et al*. [58] reported first-in-human images of coronary arteries obtained using a hybrid IVUS-OCT imaging system, the Novasight HybridTM system. This imaging modality combines OCT and IVUS, compensating for the deficiencies between the two imaging techniques. During imaging, the OCT resolution and IVUS depth can be obtained simultaneously. This enables the acquisition of high-resolution details of the vascular intima and an understanding of the overall morphology of the vessel wall and lumen, thus allowing for a more comprehensive assessment of vascular lesions.

#### 5.1.3 NIRF

NIRF is a promising visualization technique for tissue perfusion imaging. By using fluorescent probes targeting specific components in atherosclerotic plaques, such as inflammatory cells and lipids, NIRF imaging can assist doctors in more accurately assessing the nature and stability of the plaques and predicting the risk of plaque rupture and cardiovascular events [59]. However, because biological tissues themselves may generate autofluorescence, this interferes with the results of NIRF imaging, affecting the quality of the images and the accurate diagnosis of the lesions. In addition, the penetration depth of NIRF imaging is relatively shallow, and it may not be able to clearly image deeper coronary artery lesions, limiting its application in some situations [60]. In the NIRF spectral range, it is practical to measure fluorescence for perfusion assessment because of the low autofluorescence of tissues, which facilitates the visualization of intravenously injected fluorophores. Among the fluorescent dyes used for perfusion assessment, indocyanine green is the most common and is retained in the vascular system mainly because of its ability to selectively bind to albumin in plasma proteins [61]. NIRF aims to insert fibronectin outer region B in the process of angiogenesis in plaque, so as to recognize neovascularization in plaque [62]. This technology combined with OCT can make full use of the advantages of high spatial accuracy of OCT to better display the neovascular structure in the plaque [63]. This combined technique provides a powerful tool for accurately evaluating neovascularization in atherosclerotic plaques and is expected to promote an in-depth understanding of plaque vulnerability. The NIRF technique is a minimally invasive procedure with low risk of side effects. The use of NIR fluorescence imaging as a technique to assess tissue perfusion has been intensively explored in several fields.

### 5.2 Non-Invasive Detection Technology of Neovascularization in Plaque

At present, non-invasive imaging methods for neovascularization in plaques include computed tomography angiography (CTA), magnetic resonance imaging (MRI), and contrast-enhanced ultrasound (CEUS) [64]. Molecular imaging, a form of medical imaging, is capable of providing detailed information about *in vivo* physiological processes. Imaging agents are indispensable in the application of non-invasive detection techniques, as they can make physiological activities visible.

#### 5.2.1 CTA

The basic principle of CTA is based on the attenuation properties of X-rays that are absorbed and attenuated by different tissues as they pass through the body. This depends on factors such as the density and atomic number of the tissues. For example, bone, which contains many dense materials such as calcium, attenuates X-rays to a high degree, whereas air, which has a very low density, attenuates X-rays to a very low degree [65]. CT uses ion radiation and different X-ray attenuations of the body tissue to reconstruct the image of the carotid artery and its vascular wall. The advantages of CTA are its non-invasiveness and fast scanning speed. It can display the morphology, structure, and lesions of the blood vessels, including vascular stenosis, occlusion, aneurysm, and vascular malformation. In clinical applications, it can initially assess the degree and scope of vascular lesions, thereby providing a basis for the formulation of treatment plans. However, for some small blood vessels, such as those with a diameter of less than 1 mm, the display effect of CTA may not be satisfactory. Moreover, calcification of the vessel wall affects the judgment of the degree of vascular stenosis by CTA, which may lead to overestimation or underestimation of the severity of the lesions [66]. In the process of CTA, a small part of the contrast medium enters the neovascularization of the vascular wall from the arterial cavity. The CT value of the vascular wall is directly proportional to the spatial density of the neovascularization. This imaging technique can provide detailed visualization of neovascularization in the carotid artery and important information for the evaluation of atherosclerotic plaques.

#### 5.2.2 MRI

MRI generates images using a strong magnetic field and radiofrequency pulses. It has no ionizing radiation and is capable of clearly distinguishing the boundaries between the blood vessel wall, the soft tissues around the blood vessels, as well as between the lesions and normal tissues. It is of great value in detecting lesions of the blood vessel wall, such as in the compositional analysis of atherosclerotic plaques (distinguishing between lipids, fibrous tissues, calcifications, etc.). In clinical practice, cardiac MRI can accurately measure parameters such as the size of the heart, thickness of the ventricular wall, and systolic and diastolic functions of the myocardium, which is of great significance for the diagnosis and differential diagnosis of cardiomyopathies [67]. However, the scanning time for MRI is usually relatively long. Slight movements during the examination process, such as breathing, heartbeat, and swallowing, may lead to a decline in the image quality, causing artifacts and affecting the accurate judgment of vascular lesions [68]. The combination of MRI and contrast agent forms dynamic contrast-enhanced MRI (DCE-MRI), which can evaluate the structural and functional characteristics of neovascularization in plaques [69]. This method provides a non-invasive means for studying the physiological and pathological processes of neovascularization and has potentially important applications in the evaluation of atherosclerosis and other diseases. A variety of molecular magnetic resonance probes have been developed to improve the non-invasive detection and characterization of atherosclerotic plaques [70]. Among them, specifically targeted molecular probes have the unique advantage of clearly visualizing the key biological steps in the development of atherosclerotic lesions in the arterial vascular wall, opening a window into the mechanisms of the lesions. Thus, not only can the process of atherosclerosis development be detected at an early stage, but vulnerable atherosclerotic plaques can also be accurately identified, providing an important basis for subsequent diagnosis and treatment.

#### 5.2.3 CEUS

CEUS is different from other examination methods such as CTA and MRI. The examination is relatively simple and can be performed at the bedside. It enables real-time and dynamic observation of the blood flow perfusion of tissues and organs and can clearly display the course, branches of blood vessels, and dynamic changes in blood flow. Ultrasonic contrast is a type of microbubble contrast agent, mainly composed of air or inert gas, which is excreted out of the body through pulmonary respiration. In clinical practice, it can be used to evaluate the viability of patients with myocardial infarction, myocardial perfusion in cardiomyopathy, and hemodynamic changes in valvular heart diseases. However, the quality of ultrasonic contrast images is affected by various factors, such as the patient’s body shape, gas interference (such as gastrointestinal gas), and the depth of the examination site. These factors may lead to an unclear display of the ultrasonic images, thus affecting the diagnostic results [71]. CEUS has been applied for the identification of neovascularization in atherosclerotic plaques. B-ultrasound provides a two-dimensional black-and-white cross-sectional image of the target area, and the contrast agent is a microbubble filled with echo developing gas injected into the circulation system to illuminate blood flow. In neovascularized plaques, the microbubbles that reflect sound waves through the vascular wall and neovascularization in the plaque [72] generate bright areas in the newly generated microvascular aggregation areas in the plaque, in which the microbubbles move within the plaque, resulting in enhanced areas in the plaque. Visualization of neovascularization can provide important insights into plaque structure, which can assess the location and density of neovascularization [73]. Contrast-induced plaque enhancement can qualitatively and quantitatively evaluate neovascularization in the plaque according to the presence or absence of contrast agent in the plaque. This makes CEUS a valuable tool for the study of angiogenesis in atherosclerotic plaques.

### 5.3 Artificial Intelligence

Artificial intelligence can handle and analyze massive amounts of medical data. By analyzing imaging examination data, such as IVUS, OCT, and CTA, artificial intelligence (AI) systems can quickly identify subtle changes in the vessel wall and the formation of early plaques as well as distinguish different plaque types, including neovascularization within the plaques [74]. This helps detect signs of atherosclerosis before symptoms appear, enabling early intervention and treatment and reducing the risk of cardiovascular diseases.

## 6. Current Prevention and Treatment Strategies of Neovascularization in Plaque

### 6.1 Statins

Recently, statins have been associated with multiple pleiotropic effects, independent of their ability to reduce LDL-C levels [75]. This effect has also been observed in endothelial cells. In a study by Weis *et al*. [76], atorvastatin (a common statin) was found to be a significant inhibitor of endothelial cell proliferation, migration, and differentiation *in vitro*. This anti-angiogenic phenotype results in reduced VEGF expression and increased endothelial cell apoptosis. This result was also observed in a mouse model. The high concentration (2.5 mg⋅kg^-1^⋅d^-1^) significantly reduced inflammation-induced angiogenesis. Subsequent studies have shown that statins exhibit a dose-dependent relationship with angiogenesis. Low doses promote angiogenesis, whereas high doses (with stronger clinical relevance) inhibit angiogenesis [77].

### 6.2 Angiogenesis Inhibitors

Currently, more than 80 angiogenesis inhibitors are being evaluated in clinical trials [78]. For example, Bergers *et al*. [79] showed that tumor regression can be achieved in a mouse model of pancreatic cancer using four different angiogenesis inhibitors. These four inhibitors have different targets in the angiogenesis pathway: (I) small molecule inhibitors of endothelial cell proliferation; (II) MMP inhibitors that prevent endothelial cell migration during neovascularization; (III) endostatin, which inhibits angiogenic factors such as VEGF and fibroblast growth factor (FGF); (IV) angiostatin, which may inhibit angiogenesis by inhibiting the migration and proliferation of endothelial cells and inducing apoptosis. Angiogenesis inhibitors can specifically inhibit tumor angiogenesis or neovascularization within plaques, thereby restricting tumor growth and metastasis or stabilizing atherosclerotic plaques. In addition to their lipid-lowering effects, statins possess multiple effects such as anti-inflammatory properties and plaque stabilization. When these two types of drugs are used in combination, they can exert their effects at different stages, producing a synergistic effect and inhibiting disease progression more effectively [16, 17].

### 6.3 Therapeutic Contrast-Enhanced Ultrasound and Modern Nanotechnology

Contrast-enhanced ultrasound was used to observe the contrast microbubbles on the adherent target tissue. If therapeutic drugs are contained in the microbubble shell or core, it is possible to target the local delivery of these drugs. A “microcarrier system” encapsulating drugs can inject drugs into the blood of diseased vessels containing vulnerable atherosclerotic plaques and be used as targeted therapy [80]. Relevant studies have shown that angiogenesis inhibitors can be delivered to the tumor microvascular system through contrast agent microbubbles to inhibit angiogenesis [81]. Yuan *et al*. [82] found in a study of Apolipoprotein E (ApoE)-deficient mice that microbubbles were coupled with the angiogenesis inhibitor Endostar and an anti-intercellular adhesion molecule 1 (ICAM-1) antibody. Compared with the control group, the plaque area in the treatment group was significantly reduced, indicating that angiogenesis was successfully inhibited and the plaque subsided. Nanomedicine, which applies nanotechnology to the treatment, diagnosis, monitoring, and control of biological systems, represents a novel medical tool for pathologies, such as atherosclerosis. It has the ability to overcome the barriers associated with traditional drug delivery methods. The inherent biocompatibility and biodegradability of materials employed in nanomedicine render this technology particularly appealing for *in vivo* applications [83]. Notably, research on macrophage cultures involving the use of phosphatidylserine-bearing nanostructures in combination with curcumin, an anti-inflammatory and antioxidant agent, indicates that these nanostructured lipid carriers can effectively inhibit lipid accumulation and the production of pro-inflammatory factors, while also promoting the release of anti-inflammatory cytokines [84]. This intervention indirectly retards the progression of plaque while alleviating the inflammation present within the plaque.

### 6.4 Melatonin

In gastric ulcers, skin lesions, and some physiological processes, melatonin inhibits angiogenesis in tumors, age-related eye diseases, and hypoxic environments. Studies have speculated that melatonin may inhibit neovascularization in atherosclerotic plaques [85], thus preventing the occurrence and development of atherosclerosis. In addition, a study has pointed out that these effects of melatonin are related to its role in regulating vascular endothelial growth factor and its receptor [86], but the specific regulatory mechanism is still different; therefore, the effect of melatonin on angiogenesis is different under different conditions.

### 6.5 Gene Therapy and Stem Cell Therapy

In the context of atherosclerosis, an increasing number of circulating microRNAs (miRNAs) have been identified, and their levels are correlated with arterial wall thickening in the early disease stages. Current research has focused on exploring the clinical applications of specific miRNA levels as biomarkers of disease onset and progression. Based on these findings, miRNA mimics and miRNA antagonists have been employed to counteract the functional pathological consequences of decreased or upregulated miRNA expression, thereby mitigating plaque progression and suppressing intraplaque neovascularization [87]. Mesenchymal stem cells (MSCs), by virtue of their tissue repair, anti-inflammatory, and immunomodulatory properties, have proven to be effective in the treatment of various diseases, including atherosclerosis. Previous studies have shown that MSCs can regulate various inflammatory cells, including macrophages, and hinder plaque formation by suppressing inflammatory responses. Moreover, MSCs also play a role in inhibiting neovascularization within plaques by suppressing inflammation [88].

### 6.6 Diet and Exercise

Diet and exercise play crucial roles in controlling atherosclerotic plaques. The occurrence and development of atherosclerosis are closely associated with dyslipidemia. Reducing the intake of saturated fatty acids and cholesterol, such as by eating less animal offal, fatty meat, and whole-fat dairy products, can lower the level of LDL-C in the blood and reduce the deposition of lipids on the vascular wall [89]. In addition, regular exercise can enhance the body’s metabolic level, increase the oxidation and decomposition of fat, reduce the levels of triglycerides and LDL-C, and simultaneously increase the HDL-C content. Aerobic exercises, such as brisk walking, running, swimming, and cycling, can enhance cardiopulmonary function, promote blood circulation, facilitate the metabolism and transportation of lipids, reduce the deposition of lipids on the vascular wall, and thus control the progression of atherosclerotic plaques [90].

## 7. Conclusions

A large amount of scientific evidence shows that there is a clear correlation between plaque neovascularization and the growth of atherosclerotic plaques and the development of an unstable plaque phenotype, leading to plaque rupture and related clinical events. Under physiological conditions, neovascularization causes oxygen and nutrients to enter the blood vessel walls. However, when the plaque expands due to pathological stimulation, neovascularization in the plaque creates favorable conditions for plaque growth and becomes a channel for cholesterol, inflammatory cells, red blood cells, temporary extracellular matrix, or other atherosclerotic molecules to enter the growing plaque. It has been proven that preventing the growth of pathological neovascularization or maintaining existing physiological nutrient vessels can slow down the development of plaque and enhance its stability. Although these associations have been clearly identified, there is still a knowledge gap regarding the exact mechanism of the regulation of pathological neovascularization. The increased risk of neovascularization and poor cardiovascular prognosis emphasize the lack of available diagnostic and therapeutic tools in the clinic. Further research and the development of quantitative diagnostic techniques are necessary to provide more accurate plaque risk assessments. New targeted therapies for atherosclerosis, which continue to be studied, may also completely alter the treatment of this disease.

## Abbreviations

HIF1, hypoxia inducible factor 1; VEGF-A, vascular endothelial growth factor A; Ets-1, E26 transformation related factor-1; PPARγ, peroxisome proliferator-activated receptor γ; TC, total cholesterol; LDL-C, low-density lipoprotein cholesterol; non-HDL-C, non-high-density lipoprotein cholesterol; HDL-C, high-density lipoprotein cholesterol; NLRP3, nucleotide-binding oligomerization domain-like receptor family, pyrin domain-containing 3; IL-1β, interleukin 1β; MMP, matrix metalloproteinases; CEUS, contrast-enhanced ultrasound; PCI, percutaneous coronary intervention; MACE, major adverse cardiovascular events; IVUS, intravascular ultrasound; OCT, optical coherence tomography; NIRF, near-infrared fluorescence; CTA, computed tomography angiography; MRI, magnetic resonance imaging; DCE-MRI, dynamic contrast-enhanced MRI; FGF, fibroblast growth factor; ICAM-1, intercellular adhesion molecule 1; miRNAs, microRNAs; MSCs, mesenchymal stem cells.
